# A toolkit for rapid gene mapping in the nematode *Caenorhabditis briggsae*

**DOI:** 10.1186/1471-2164-11-236

**Published:** 2010-04-13

**Authors:** Daniel C Koboldt, Julia Staisch, Bavithra Thillainathan, Karen Haines, Scott E Baird, Helen M Chamberlin, Eric S Haag, Raymond D Miller, Bhagwati P Gupta

**Affiliations:** 1 Department of Genetics, Washington University School of Medicine, St. Louis, Missouri 63110, USA; 2 Department of Biology, McMaster University, Hamilton, ON L8S 4K1, Canada; 3 Department of Biological Sciences, Wright State University, Dayton, OH 45435, USA; 4 Department of Molecular Genetics, Ohio State University, Columbus, OH 43210, USA; 5 Department of Biology, University of Maryland, College Park, MD 20742, USA

## Abstract

**Background:**

The nematode *C. briggsae *serves as a useful model organism for comparative analysis of developmental and behavioral processes. The amenability of *C. briggsae *to genetic manipulations and the availability of its genome sequence have prompted researchers to study evolutionary changes in gene function and signaling pathways. These studies rely on the availability of forward genetic tools such as mutants and mapping markers.

**Results:**

We have computationally identified more than 30,000 polymorphisms (SNPs and indels) in *C. briggsae *strains AF16 and HK104. These include 1,363 SNPs that change restriction enzyme recognition sites (snip-SNPs) and 638 indels that range between 7 bp and 2 kb. We established bulk segregant and single animal-based PCR assay conditions and used these to test 107 polymorphisms. A total of 75 polymorphisms, consisting of 14 snip-SNPs and 61 indels, were experimentally confirmed with an overall success rate of 83%. The utility of polymorphisms in genetic studies was demonstrated by successful mapping of 12 mutations, including 5 that were localized to sub-chromosomal regions. Our mapping experiments have also revealed one case of a misassembled contig on chromosome 3.

**Conclusions:**

We report a comprehensive set of polymorphisms in *C. briggsae *wild-type strains and demonstrate their use in mapping mutations. We also show that molecular markers can be useful tools to improve the *C. briggsae *genome sequence assembly. Our polymorphism resource promises to accelerate genetic and functional studies of *C. briggsae *genes.

## Background

Comparative analysis of developmental and behavioral processes in closely related species is a powerful approach to understand the mechanisms of evolution. It facilitates identification of molecular components that are conserved over millions of years due to their role in specifying common features as well as those that are variable because they confer species-specific features. The model organism *Caenorhabditis elegans *(a nematode) and its congener, *C. briggsae*, are particularly suitable for such investigations. Their many experimental advantages include rapid growth, small size, transparency, ease of culture and genetic manipulations, and the availability of fully sequenced genomes [1-3].

*C. briggsae *is phenotypically almost indistinguishable from *C. elegans *and has a similar (hermaphroditic) reproductive mode. The last common ancestor of these two species lived about 30 million years ago [4], and despite the rapid molecular evolution typical of the family Rhabditidae, more than half (~52%) of the *C. elegans *genome aligns with the *C. briggsae *genome assembly [2]. This includes two-thirds of all *C. briggsae *genes (13,107 or 67.8%) with reciprocal orthologs in *C. elegans *[5]. Thus *C. elegans-C. briggsae *comparative genomic and genetic studies promise powerful new tools for the identification of genes and pathways and the study of both conservation and divergence.

Like *C. elegans*, *C. briggsae *has six chromosomes that display extensive conservation of synteny, but not exact colinearity relative to *C. elegans *[6]. While *C. briggsae *shares many of the experimental advantages of *C. elegans*, it has the further advantage of increased natural variability for single nucleotide polymorphisms (SNPs) and insertion-deletions (indels) [7,8]. This elevated natural variation potentially enhances its use for genotype-phenotype association studies, and is also very useful for the mapping aspects of forward genetics projects.

Initial work on gene function in *C. briggsae *employed cross-species transgene rescue of *C. elegans *mutants (e.g. [9-13]) and RNA interference (RNAi; e.g. [13-17]). However, a number of laboratories are now generating true mutations in *C. briggsae*, using both forward mutagenesis screens [18-20] (R.E. Ellis, personal communication; B.P. Gupta, unpublished results; H. Chamberlin, unpublished results) and PCR-based deletion mutation screens [20]. Positional cloning of *C. briggsae *mutations without relying upon obvious candidate genes requires a set of mapping tools. Development of such tools is facilitated by a high-quality whole-genome shotgun assembly [2] and the organization of most of the resulting contigs into chromosomes via a SNP-based recombination map [6].

Among the tools needed to facilitate forward genetics in *C. briggsae*, a set of easily scored DNA polymorphisms is especially important. Experimentally validated polymorphisms can serve as useful markers for mapping mutations that cause visible phenotypes. Additionally, these markers can be integrated with the phenotype-based genetic linkage map (e.g., *dpy *and *unc *mutants [21]) to further enhance their utility. Integration of polymorphisms and phenotype-based maps increases map density and anchors the relative locations of molecular and phenotypic markers. With this goal in mind we have discovered a large set of genome-wide polymorphisms (SNPs and indels) in wild-type strains, using AF16 as a reference strain and four other natural isolate strains: HK104, HK105, VT847, and PB800.

The indels were placed into three classes: small (7-49 bp), medium (50-2,000 bp), and large (>2 kb). We have focused on medium and small indels (212 and 7,530, respectively), which offer the greatest utility as genetic markers. In the case of SNPs (23,829), we found that 4,700 modify restriction enzyme sites (termed snip-SNPs) and therefore can be easily detected as restriction fragment length polymorphisms (RFLPs). We established assay conditions for bulk segregant analysis (BSA) and used these to experimentally validate 14 snip-SNPs, 28 medium and 32 small indels. The validated polymorphisms were used to genetically map known mutations causing visible phenotypes thus demonstrating the effectiveness of the polymorphisms in linkage mapping studies. We also developed single animal-based PCR assay to determine map distance. Five mutations were successfully localized to sub-chromosomal regions by 3 or more indels, greatly facilitating the search for each candidate gene. These results demonstrate the utility of our mapping toolkit in genetic linkage and gene identification studies.

## Results

### SNP Discovery

**Figure 1:**
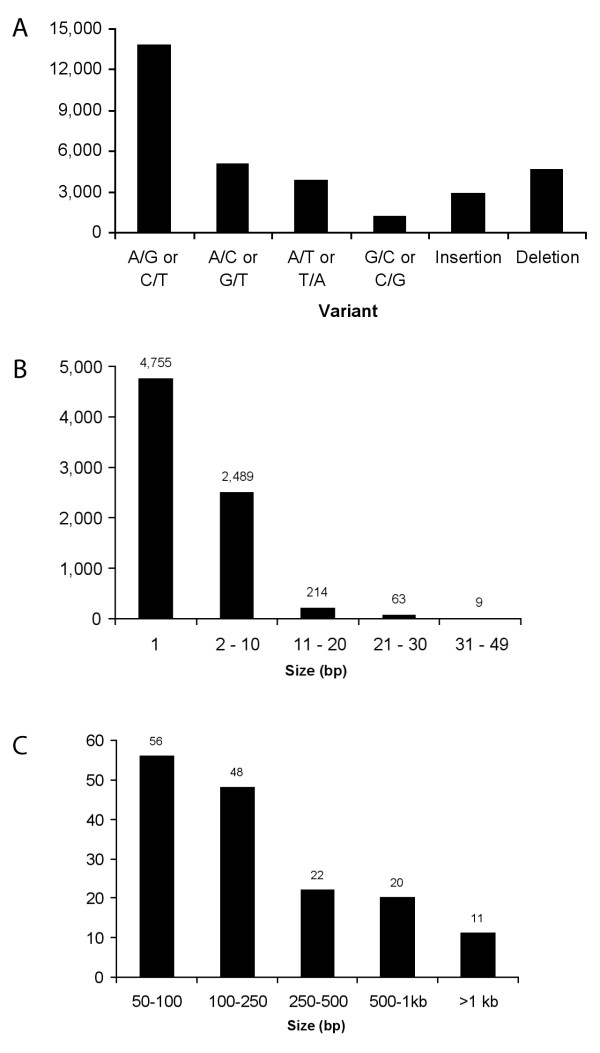
**SNP discovery results for *C. briggsae *strain HK104**. (A) Incidence of SNPs and small indels identified by ssahaSNP, by polymorphism type. (B) Distribution of small (<50 bp) indels, by size. (C) Distribution of moderate (50-2,000 bp) indels, by size.

We performed SNP discovery in four *C. briggsae *strains by aligning paired shotgun sequence reads to the AF16-based reference sequence (cb25 assembly, [22]). These sequences were obtained by capillary gel electrophoresis at Washington University Genome Center (see Methods). To build on previous SNP discovery efforts [6], we applied the ssahaSNP algorithm, which detects SNPs and small indels based on SSAHA alignments to a reference sequence (see Methods). Compared to AF16, ssahaSNP detected 23,829 unique SNP loci in HK104 DNA, or one substitution per 163 bp on average (Table 1 and additional file 1). Consistent with *C. briggsae *clade structure [8], SNP density was slightly lower in strains HK105 (1/168 bp) and PB800 (1/197 bp) and much lower in strain VT847 (1/475 bp). In HK104, the most common substitution by far was A(T) to G(C), which accounted for 57.1% of all substitutions (Figure 1A).

**Table 1:** SNPs in various *C. briggsae *strains identified by ssahaSNP (in comparison to AF16).

	**HK104**	**VT847**	**HK105**	**PB800**
	
Sequence traces examined	13,632	14,976	2,112	384
Traces aligned by SSAHA	7,530	9,213	1,680	123
Total aligned base pairs	4,562,172	5,761,972	1,038,254	75,508
Total unique aligned base pairs	3,884,127	4,327,725	867,552	63,434
Unique SNP loci detected	23,829	9,111	5,164	322
Apparent SNP density (per kb)	6.13	2.11	5.95	5.08

Total aligned base pairs include redundant matches due to sequence overlaps (between 15% and 25%) in sequence data. The SNP density is based on the number of uniquely aligned base pairs.

### RFLP genotyping assays and validation of snip-SNPs

We screened the SNPs predicted for HK104 for variants that altered the recognition site of a restriction enzyme, and thus might be amenable to restriction fragment length polymorphism (RFLP) genotyping. To make this a practical resource, we limited the analysis to 30 restriction enzymes from REBASE [23] that are reliable and inexpensive. Of 23,829 HK104 SNPs, some 4,700 (19.72%) were predicted to alter the recognition site of at least one of the 30 restriction enzymes. To develop restriction fragment length polymorphism (RFLP) assays from these snip-SNPs, we designed PCR primers with a standard protocol and performed *in silico *digests of the resulting amplicons to infer the banding patterns for each strain. RFLPs not easily distinguishable on a gel, or SNPs on ultracontigs not yet included in the genetic map, were removed. Finally, we used assembly AGP information and BLAST alignment to obtain coordinates for each snip-SNP on the cb3 sequence assembly. Our set contained 1,987 predicted RFLP assays from 1,362 snip-SNPs (some SNPs alter multiple RE sites) positioned on both the genetic and physical maps (see additional file 2). Another snip-SNP, bdP3, was identified in a separate study (see Methods and additional files 1 and 2).

**Figure 2:**
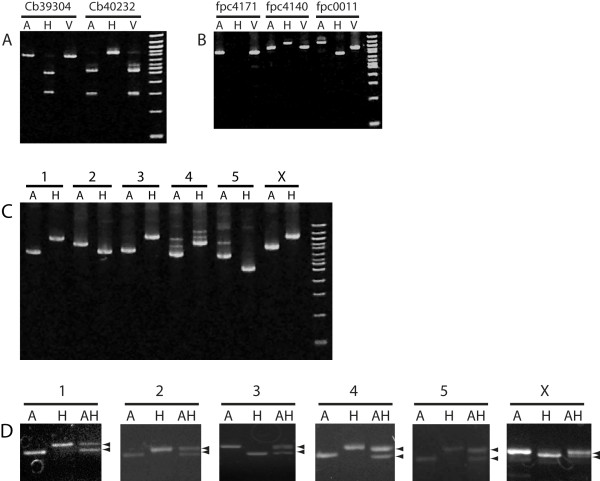
**Validation of polymorphism assays in *C. briggsae***. (A) RFLP assays for snip-SNPs in three parental strains, namely AF16 (A), HK104 (H), and VT847 (V) by *Hind*III restriction digestion. (B) Medium indels in three parental strains showing a 100 bp deletion on *fpc4171*, a 145 bp insertion on *fpc4140*, and a 536 bp deletion on *fpc0011*. (C) Six additional medium indels (one for each chromosome) in AF16 and HK104 genotypes. The indels are (Chr. 1-5 and X, from left to right): cb-m142 (250 bp), cb-m21 (200 bp), cb-m205 (300 bp), cb-m172 (250 bp), cb-m103 (200 bp), and cb-m204 (250 bp). (D) Validation of small indels in AF16, HK104 and F1 heterozygotes (AH). Chr 1: bhP19 (32 bp), Chr 2: bhP21 (20 bp), Chr 3: bhP12 (39 bp), Chr 4: bhP9 (16 bp), Chr 5: bhP48 (22 bp) and Chr X: bhP24 (16 bp).

We selected a total of 20 RFLP assays (between 3 and 4 for each chromosome) based on *Hind*III, *Dra*I and *Sal*I snip-SNPs for validation in AF16 and HK104 parental DNA (Table 2). Roughly a third of the assays (6) failed PCR in one or both strains in repeated attempts. Although we did not investigate the issue of PCR failure, it is possible that redesigning primers (by moving them out or in) and testing different PCR conditions may produce desired products in some cases. All of the 14 assays successfully gave rise to strain-specific RFLP banding patterns, validating the predicted snip-SNP (Table 2, Figure 2A for two examples). Interestingly, two of these assays (cb55670 and cb20723) exhibited HK104 fragments that varied from *in silico *predictions, another possible consequence of unknown variants in this highly divergent strain. Consistent with *C. briggsae *clade structure [8], VT847 was not polymorphic (from AF16) for the snip-SNPs we examined.

**Table 2:** List of validated snip-SNPs by RFLP assays.

Chr	SNP id	Location (cM)	Ultra_contig	RE	DNA fragments in AF16 (bp)		DNA fragments in HK104 (bp)		Validation type
					
					Expected	Observed	Expected	Observed	
1	cb15251	13.45	cb25.fpc4321	DraI	747	700	332 and 415	310 and 400	*
	cb55627	21.82	cb25.fpc3441	HindIII	691	691	251 and 440	270 and 440	*
	cb55670	21.82	cb25.fpc3441	DraI	312 and 437	749	749	312 and 437	**
	cb650	43.93	cb25.fpc4140	SacI	689	689	257 and 432	257 and 432	*
	(bhP27)								

2	cb41028	11.88-13.1	cb25.fpc0011	DraI	332 and 419	332 and 400	751	800	*
	cb43091	21.28-26.58	cb25.fpc0058	HindIII	748	800	365 and 383	383 and 383	*
	cb64777	27.92-33.95	cb25.fpc4206	DraI	325 and 424	325 and 400	749	749	*

3	cb20723	12.46	cb25.fpc4153	DraI	20, 196, 220 and 311	230, 311 and 390	20, 196 and 531	230 and 800	**
	cb54953	21.77	cb25.fpc2976	HindIII	751	800	368 and 383	380 and 400	*
	cb40003	31.14	cb25.fpc0002	DraI	685	PCR failure	208 and 477	800	

4	cb8971	7.88	cb25.fpc4250	DraI	750	PCR failure	337 and 413	350 and 413	
	cb48850	20.02	cb25.fpc1570	HindIII	750	780	314 and 436	350 and 500	*
	cb56202	37.52-41.16	cb25.fpc3835	DraI	60, 190 and 489	60, 190 and 500	60 and 679	60 and 800	*

5	cb39304	20.98-22.23	cb25.fpc4126	HindIII	742	790	286 and 456	310 and 470	*
	cb39354	20.98-22.23	cb25.fpc4126	DraI	751	PCR failure	269 and 482	PCR failure	
	cb62	17.07-17.74	cb25.fpc4470	DraI	54, 295 and 400	PCR failure	54 and 695	800	
	bdP3	unknown	cb25.fpc0156	DraI	907	907	227 and 680	227 and 680	*

x	cb20148	9.52	cb25.fpc0045	DraI	660	PCR failure	171 and 489	PCR failure	
	cb40232	20.1-20.71	cb25.fpc0003	HindIII	237 and 473	300 and 473	710	850	*
	cb6050	24.07	cb25.fpc4403	DraI	750	PCR failure	270 and 480	290 and 510	

The snip-SNPs are arranged by chromosome and location. The corresponding ultracontigs are also listed. The expected and observed DNA fragments refer to products based on *in silico *analysis and actual experiments, respectively. The bdP3 location is unknown because the corresponding contig is unassembled. The validation type column marks snip-SNPs that were consistent with prediction (*) and those that differed significantly (**). RE: Restriction enzyme used to digest PCR amplified products.

### Indel Discovery

The ssahaSNP program [24] was also able to detect insertion/deletion (indel) polymorphisms of 1-50 bp (Figure 1B). We used the *parse_indel *utility to extract 7,530 candidate indels (4,686 deletions and 2,844 insertions compared to AF16) for the HK104 strain. Most insertions and deletions detected by ssahaSNP were single base pair events; the largest was 49 bp. To identify larger insertion/deletion events, we developed a customized algorithm called BreakPointRead that detects indels based on BLAST alignments between read sequences and the reference genome. First, the algorithm identifies "breakpoint reads" with alignment gaps of 10 bp or larger compared to the reference sequence. Next, it analyzes the gap size and alignment orientations to infer the nature (insertion, deletion, inversion, etc.) and approximate size of sequence variation. When we applied BreakPointRead to the HK104 sequence traces, it identified 689 breakpoint reads suggestive of 635 underlying variants. We filtered the results to obtain insertion/deletion events between 50 and 2,000 bp. The resulting set contained 212 predicted indels (144 deletions and 68 insertions), the largest of which was a 1,707 bp deletion on chromosome IV (Figure 1C and additional file 3).

### Validation of indels

#### Medium indels

We used the BreakPointRead algorithm to develop PCR fragment length polymorphism (PLP) assays for medium indels to facilitate high-throughput gene mapping. Of the 212 putative indels, we selected 40 for validation in AF16 and HK104 parental DNA (Figure 2B, C). Two other indels (bdP1 and bdP4) were also chosen for a similar analysis (see Methods and additional file 3). Ten of the 42 PLP assays failed PCR in one or both strains (Table 3). As mentioned earlier, some of these errors may be eliminated by redoing PCR using new primers. One assay (cb-m16) resulted in PCR products that were quite large (>1,500 bp) in both strains and therefore could not be accurately resolved on the gel. Of the remaining 31 assays that were successfully amplified, 29 confirmed the presence of polymorphism, 22 of which were similar in type and size as predicted (Table 3).

**Table 3:** List of medium indels tested by PCR.

Chr	Indel ID	Location	Ultra_contig	AF16 Amplicon (bp)		HK104 Amplicon (bp)		Predicted indel	Validation status	Validation Type
								
		(cM)		Predicted	Observed	Predicted	Observed			
1	cb-m142	12.6	cb25.fpc4321	665	700	915	950	250 bp insertion	250 bp insertion	*
	cb-m2	13.5	cb25.fpc4321	1461	Failed PCR	1125	Failed PCR	336 bp deletion	Failed PCR	
	cb-m5	20.0	cb25.fpc4171	774	750	674	Failed PCR	100 bp deletion	Failed PCR	
	cb-m5	20.0	cb25.fpc4171	461	500	361	500	100 bp deletion	no indel	
	cb-m6	43.9	cb25.fpc4140	930	900	785	1000	145 bp deletion	100 bp insertion	**
	cb-m13	unknown	cb25.fpc0078	504	420	385	400	119 bp deletion	20 bp deletion	**
	cb-m14	unknown	cb25.fpc4127	1000	1000	676	710	324 bp deletion	290 bp deletion	*
	cb-m146	unknown	cb25.fpc4122	406	480	506	530	100 bp insertion	50 bp insertion	**
	cb-m12	unknown	cb25.fpc4127	921	Failed PCR	416	1000	505 bp deletion	Failed PCR	

2	cb-m21	24.2	cb25.fpc0058	825	900	670	700	155 bp deletion	200 bp deletion	*
	cb-m16	37.4	cb25.fpc4071	1097	>1500	687	>1500	410 bp deletion	unclear	
	cb-m19	11.9-13.1	cb25.fpc0011	1364	1013	828	750	536 bp deletion	263 bp deletion	**
	cb-m149	27.9-34.0	cb25.fpc4206	470	490	603	600	133 bp insertion	110 bp insertion	*
	cb-m26	27.9-34.0	cb25.fpc4206	678	700	407	410	271 bp deletion	290 bp deletion	*
	cb-m38	unknown	cb25.fpc4131	835	860	544	600	291 bp deletion	260 bp deletion	*
	bdP4	unknown	cb25.fpc2441	1013	~990	903	~880	110 bp deletion	110 bp deletion	*

3	cb-m46	6.1	cb25.fpc2587	747	1020	574	750	173 bp deletion	270 bp deletion	*
	cb-m155	6.1	cb25.fpc2587	1169	Failed PCR	1329	Failed PCR	160 bp insertion	Failed PCR	
	cb-m48	21.2	cb25.fpc2976	1163	1200	644	1200	519 bp deletion	no indel	
	cb-m205	25.8	cb25.fpc4079	671	700	971	1000	300 bp insertion	300 bp insertion	*
	cb-m159	36.5	cb25.fpc4224	463	500	655	Failed PCR	192 bp insertion	Failed PCR	
	cb-m160	28.8-29.3	cb25.fpc2193	821	1000	1053	1200	232 bp insertion	200 bp insertion	*
	bdP1	31.3	cb25.fpc0002	1723	~1700	~1500	~1500	~200 bp deletion	200 bp deletion	*

4	cb-m172	18.2	cb25.fpc0039	650	700	904	950	254 bp insertion	250 bp insertion	*
	cb-m170	20.6	cb25.fpc4090	691	720	931	Failed PCR	240 bp insertion	Failed PCR	
	cb-m171	25.5	cb25.fpc4132	381	450	476	550	95 bp insertion	100 bp insertion	*
	cb-m70	25.5	cb25.fpc4132	594	>1600	421	1600	173 bp deletion	very large indel	**
	cb-m176	37.5-41.2	cb25.fpc3835	732	Failed PCR	1014	Failed PCR	282 bp insertion	Failed PCR	
	cb-m74	37.5-41.2	cb25.fpc3835	677	1000	523	700	154 bp deletion	300 bp deletion	**
	cb-m76	42.4-43.0	cb25.fpc2328	709	900	527	550	182 bp deletion	350 bp deletion	**
	cb-m177	9.2-9.8	cb25.fpc4118	801	800	946	900	145 bp insertion	100 bp insertion	*
	cb-m179	unknown	cb25.fpc4331	383	450	500	520	117 bp insertion	70 bp insertion	*

5	cb-m103	19.0	cb25.fpc2220	600	700	454	500	146 bp deletion	200 bp deletion	*
	cb-m97	46.1	cb25.fpc4109	591	705	392	490	199 bp deletion	215 bp deletion	*
	cb-m105	37.0-37.7	cb25.fpc4063	692	800	454	520	238 bp deletion	280 bp deletion	*
	cb-m104	37.0-37.7	cb25.fpc4063	942	Failed PCR	661	Failed PCR	281 bp deletion	Failed PCR	

x	cb-m197	3.5	cb25.fpc4033	692	700	855	790	163 bp insertion	90 bp insertion	*
	cb-m204	17.4	cb25.fpc4044	689	750	949	1000	260 bp insertion	250 bp insertion	*
	cb-m136	30.1	cb25.fpc0829	1440	2000	645	750	795 bp deletion	1250 bp deletion	*
	cb-m127	34.1	cb25.fpc2334	486	600	349	405	137 bp deletion	195 bp deletion	*
	cb-m126	34.1	cb25.fpc2334	785	Failed PCR	648	Failed PCR	137 bp deletion	Failed PCR	
	cb-m135	27.4-34.1	cb25.fpc0829	720	900	584	Failed PCR	136 bp deletion	Failed PCR	

The indels are arranged by chromosome and location. The corresponding ultracontigs are also listed. The "unknown" locations refer to unassembled contigs. The predicted indel sizes are based on *in silico *analysis of AF16 and HK104 amplicons. The validation type column marks indels that were consistent with prediction (*) and those that differed significantly (**).

#### Small indels

We developed PLP assays for small (<50 bp) indels in AF16 and HK104 that were identified by ssahaSNP. To allow for gel resolution, we excluded indels smaller than 7 bp. This resulted in 436 assays that had 7-49 bp band size differences between AF16 and HK104 (see additional file 4). We tested 45 indels (between 4 and 9 for each chromosome) by PCR and found that except one (bhP44), for which HK104 amplification failed, all others could be successfully amplified (Table 4). A total of 32 indels showed bands of predicted sizes (Table 4, Figure 2D). Of the remaining 12, 1 showed no indel (i.e., identical PCR products in AF16 and HK104), 4 had multiple products (either due to PCR error, incorrect *in silico *predictions, or misassembly), and 7 showed PCR products that were inconsistent and unreliable (Table 4).

**Table 4:** List of small indels tested by PCR.

Chr	Indel ID	Location (cM)	Ultra_contig	Predicted indel size (bp)	Predicted amplicon		Status
						
					AF16	HK104	
1	bhP41	unknown	cb25.fpc4180	16	242	258	Inconsistent products
	bhP42	unknown	cb25.fpc4184	15	252	267	True
	bhP19	0.7	cb25.fpc2695	32	247	215	True
	bhP43	14	cb25.fpc3857b	12	245	257	Multiple products
	bhP7	28.6	cb25.fpc2032	7	246	239	True
	bhP1	29.2	cb25.fpc3441	10	250	260	True
	bhP29	~52	cb25.fpc4140	17	248	231	True

2	bhP20	2.4	cb25.fpc4168	21	248	227	Inconsistent products
	bhP6	~8	cb25.fpc0071	11	973	962	True
	bhP2	~10	cb25.fpc3052a	7	243	236	True
	bhP33	~10	cb25.fpc3052a	44	245	201	Inconsistent products
	bhP28	11.9-13.1	cb25.fpc0011	22	249	227	Inconsistent products
	bhP32	11.9-13.1	cb25.fpc0011	21	250	271	True
	bhP21	23.3-28.6	cb25.fpc0058	20	251	231	True
	bhP44	~38	cb25.fpc1402a	12	312	324	Failed PCR in HK104
	bhP8	49.9	cb25.fpc0305	18	249	231	True

3	bhP18	0	cb25.fpc4010	8	248	256	True
	bhP14	12.5	cb25.fpc4153	22	241	219	True
	bhP38	16.8-17.5	cb25.fpc2187	44	249	293	True
	bhP12	21.2	cb25.fpc2976	29	248	219	True
	bhP34	21.8	cb25.fpc2976	7	246	239	True
	bhP39	25.7	cb25.fpc0201	13	250	263	Multiple products
	bhP40	30	cb25.fpc0002	20	250	270	True
	bhP10	35.4	cb25.fpc4224	11	249	238	No indel

4	bhP13	1.9-5.1	cb25.fpc3752	14	250	264	True
	bhP15	7.9	cb25.fpc4250	20	250	270	True
	bhP45	17.6	cb25.fpc4260	20	245	265	True
	bhP4	18.2-18.8	cb25.fpc0039	9	250	241	Inconsistent products
	bhP11	20.6	cb25.fpc4090	18	251	233	True
	bhP9	31	cb25.fpc1570	16	256	272	True
	bhP16	43.5	cb25.fpc0107	21	251	230	True
	bhP30	57.8	cb25.fpc3052b	20	248	262	Inconsistent products
	bhP46	57.8	cb25.fpc3052b	10	242	252	Multiple products

5	bhP22	1.9	cb25.fpc4095	14	249	263	Inconsistent products
	bhP31	2.5-3.2	cb25.fpc2114	24	244	268	True
	bhP47	9.6	cb25.fpc2887a	21	243	222	True
	bhP37	18.9	cb25.fpc4470	10	251	261	True
	bhP5	26.7	cb25.fpc0090	16	291	275	True
	bhP23	26.7	cb25.fpc0090	22	250	228	Multiple products
	bhP48	40.2-40.8	cb25.fpc4063	22	249	271	True
	bhP24	56.9	cb25.fpc0129	16	242	258	True

X	bhP25	8.4	cb25.fpc0045	32	250	218	True
	bhP36	13.6-16.3	cb25.fpc4044b	11	244	255	True
	bhP26	21-21.7	cb25.fpc0106	22	251	229	True
	bhP49	34.1	cb25.fpc0829	14	250	264	True

The table is organized similar to Table 3. The "Status" column shows whether an indel was correctly verified by PCR (True) or not (False). In all cases DNA sizes were determined by visual inspection.

**Figure 3:**
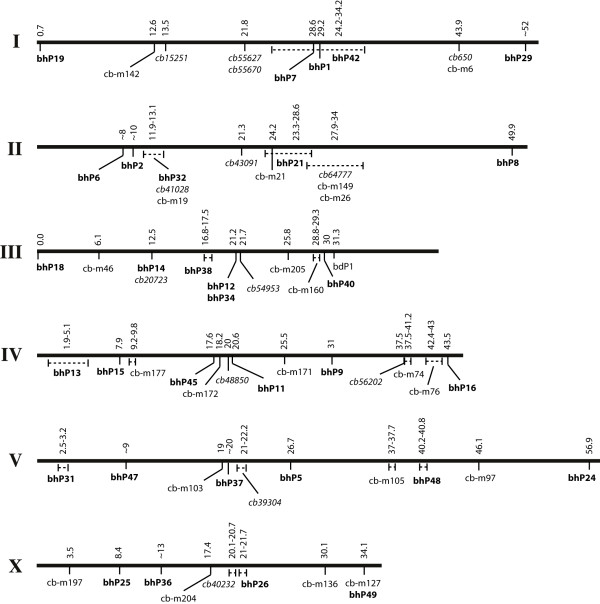
**A polymorphism-based genetic linkage map of *C. briggsae***. The map consists of 13 snip-SNPs (italics), 22 medium indels, and 32 small (bold) indels.

Altogether we experimentally confirmed 75 polymorphisms (14 snip-SNPs, 29 medium indels, and 32 small indels) on all six chromosomes (Figure 3, Table 5). The utility of these 'working' markers in genetic studies is demonstrated by successful mapping of several mutations that cause visible phenotypes. In two cases these mapping experiments also helped improve the genome sequence assembly. Specifically, the bhP42 contig *fpc4184 *was placed near the center of chromosome 1 and bhP18 contig *fpc4010 *was reassigned to the left arm of chromosome 3 (see below).

**Table 5:** Summary of polymorphisms experimentally tested in this study.

Category	Snip-SNP	Medium Indel	Small indel	Total
Attempted	20	42	45	107

PCR failure cases	6	10	1	17

Successful PCR cases	14	32	44	90
Similar	12 (86%)	22 (69%)	32 (73%)	66 (73%)
Different	2	7	0	9
False	0	3	12	15

A summary of data presented in Tables 2-4. The successful PCR cases are divided into three categories: similar (DNA fragments predicted correctly), different (DNA fragments that differed significantly from prediction), and false (no polymorphism).

#### Linkage mapping studies using snip-SNPs and indels

**Figure 4:**
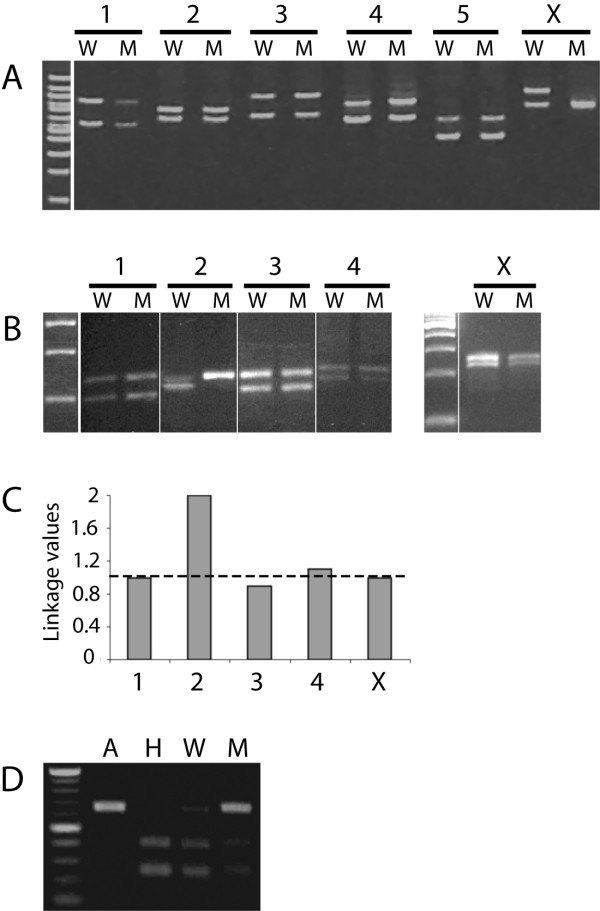
**Mutation mapping by polymorphisms**. The indels were used to map *dpy(sy5001) *(A) and *dpy(sy5148) *(B, C) and snip-SNP (cb650) was used to map *lin(bh25) *(D). (A) Mapping of X-linked mutation *dpy(sy5001) *using six medium indels (one per chromosome). W, non-mutant (phenotypically wild type) pool; M, mutant pool. (B) *dpy(sy5148) *localization on chromosome 2 by small indels (Chr 1: bhP19, Chr 2: bhP21, Chr 3: bhP12, Chr 4: bhP11 and Chr X: bhP26). (C) ULVs for *dpy(sy5148) *show linkage to bhP21. X-axis shows chromosomes whereas Y-axis linkage values. The dotted line shows the baseline for unlinked chromosomes. (D) *Sac*I digested PCR amplified genomic DNA of wild-type controls (A: AF16, H: HK104) and *lin(bh25) *mutant (M) and non-mutant (W) categories. There is a clear bias towards AF16 DNA (uncut) in the mutant pool compared to the non-mutant pool, demonstrating that *lin(bh25) *is linked to cb650 (chr. 1).

The snip-SNPs and indels were used to map a set of 12 mutations with easily recognizable phenotypes (e.g., Uncoordinated or Unc and Dumpy or Dpy) that were previously isolated in different laboratories (Table 6 and Methods). Except *lin(bh25) *and *unc(sy5415)*, all other loci were uniquely assigned to linkage groups by standard 2-factor mapping using known mutations that serve as reference (Table 6, also see [21]). The *dpy(s1272), unc(s1270)*, and *unc(sa997) *are reference markers for LGIII, LGIV, and LGV, respectively. The remaining autosomal loci are linked to *C. elegans *orthologs *Cbr-lin-11 *(LGI) and *Cbr-unc-4 *(LGII). Not only did the polymorphism-based mapping agree with phenotypic marker-based mapping (see *dpy(sy5001) *and *dpy(sy5148) *in Figure 4A, B), it also helped to identify linkage groups of *lin(bh25) *(LGI, see Figure 4D and Table 6) and *unc(sy5415) *(LGV, see Table 6). In each of these cases a single cross with HK104 provided enough genomic DNA and usually one PCR per chromosome was sufficient to establish the linkage (using bulk-segregant approach, BSA). We also quantified DNA band intensities to determine linkages as unitless linkage values (ULVs) (see Methods). As expected, for unlinked loci the ULV was one. In the case of *dpy(sy5148) *the ULV for chromosome 2 indel (*bhP21*) was 2.7 suggesting a strong linkage (Figure 4C). Overall, these results demonstrate that polymorphism-based mapping can be used to quickly map new mutations in *C. briggsae*.

**Table 6:** List of mutations used in polymorphism mapping experiments.

Mutation	Phenotype	Linkage group based on phenotypic markers	Linked chromosome and polymorphisms	Mutation source
*lev(sy5440)*	Lev-R, Unc	LGI *(Cbr-lin-11)*	1 (bhP34)	Sternberg lab
*lin(bh25)*	Egl, Lin, Unc	?	1 (bhP1, bhP29, cb650)	Gupta lab
*dpy(nm4)*	Dpy	LGII *(Cbr-unc-4)*	2 (bhP21)	Haag lab
*dpy(sy5148)*	Dpy	LGII *(Cbr-unc-4)*	2 (bhP21)	Sternberg lab
*dpy(s1272)*	Dpy	LGIII	3 (bhP12, bhP14, bhP18)	Baillie lab
*unc(sa972)*	Unc, Sma	LGIII *(dpy(s1272))*	3 (bhP14, bhP18)	Thomas lab
*lin(bh20)*	Egl, Vul	LGIII *(dpy(s1272))*	3 (bhP14, bhP38, bhP40)	Gupta lab
*unc(sy5422)*	Unc	LGIV *(unc(s1270))*	4 (bhP9, bhP11, bhP15, bhP16)	Sternberg lab
*unc(sa997)*	Unc	LGV	5 (bhP24, bhP31)	Thomas lab
*unc(sy5415)*	Unc	?	5 (bhP37)	Sternberg lab
*unc(sy5506)*	Unc	LGX	X (bhP26)	Sternberg lab
*dpy(sy5001)*	Dpy	LGX	X (bhP36, cb-m136, cb-m197, cb-m204)	Sternberg lab

Mutations are arranged by linkage group (LG) that corresponds to respective chromosome. For locations of polymorphism, refer to Figure 3. The mutant phenotypes are - Lev-R: Resistant to 1 mM levamisole; Unc: Uncoordinated; Dpy: Dumpy; Egl: Egg-laying defective; Lin: Lineage defective; Sma: Small; Vul: Vulvaless. Question marks (?) refer to mutations that were not mapped by phenotypic markers.

**Figure 5:**
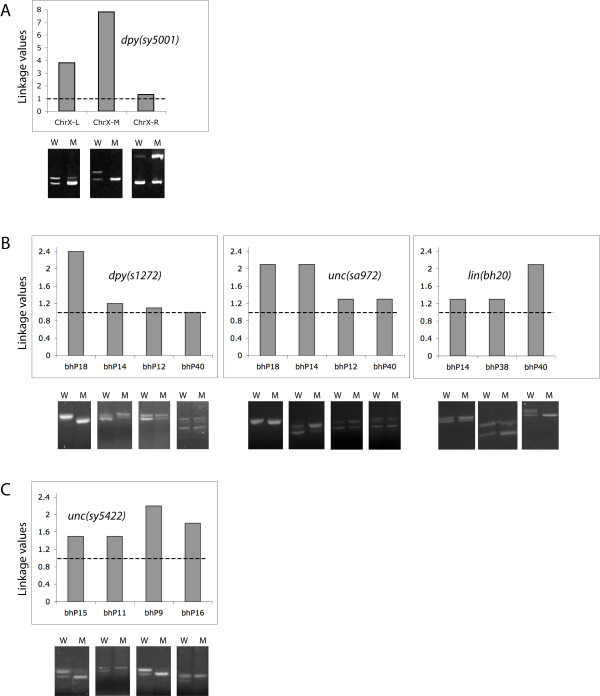
**Sub-chromosomal localization of mutations by medium and small indel-based mapping**. The gel images show non-mutant (wild type, W) and mutant (M) pool of PCR amplified DNA from F2 worms. The histograms show ULVs for various indels. (A) *dpy(sy5001) *is most strongly linked to the medium indel cb-m197 (located roughly in the middle of chromosome X, ChrX-M) compared to flanking indels cb-m204 (left arm, ChrX-L) and cb-m136 (right arm, ChrX-R). (B) *dpy(s1272), unc(sa972)*, and *lin(bh20) *are located on chromosome 3. While *dpy(s1272) *and *unc(sa972) *are strongly linked to bhP14 and bhP18 and appear to be on the left arm, *lin(bh20) *maps closer to bhP40 (center right region). (C) *unc(sy5422) *is tightly linked to indels bhP9 and bhP16 on the right arm of chromosome 4.

In addition to linking mutations to chromosomes we also investigated whether polymorphisms could be used in more precise mapping i.e., placing mutations in specific chromosomal regions (left, right arms, or middle). We reasoned that by narrowing down genetic intervals of mutations it should be possible to identify potential candidates, including *C. elegans *orthologs, thereby facilitating gene cloning by RNAi and transgene rescue approaches. To this end we used three medium indels on chromosome X to map *dpy(sy5001)*. The ULVs for *dpy(sy5001) *suggest weak linkages to indels cb-m204 (left arm) and cb-m136 (right arm) and tight linkage to the middle indel cb-m197 (Figure 5A). Similar result was also obtained with the small indel bhP26 that is located close to cb-m197 and is strongly linked to *dpy(sy5001) *(data not shown).

Besides *dpy(sy5001)*, we also mapped 4 autosomal mutations to sub-chromosomal regions using small indels. The results showed that *dpy(s1272) *is most strongly linked to bhP18, *unc(sa972) *to bhP14 and bhP18, and *lin(bh20) *to bhP40 (all on chromosome 3) (Figure 5B). The *unc(sy5422) *appears to be located on the right arm of chromosome 4 (closer to the middle) since it shows strongest linkage to bhP9 (Figure 5C).

The *C. briggsae *sequence assembly 'cb3' had placed the bhP18 contig *fpc4010 *on the right arm of chromosome 3 (~36.5 mu). We found that this location was inconsistent with ULVs for *dpy(s1272) *and *unc(sa972) *since both mutations are also linked to the left arm indel bhP14 (Figure 5B). This suggested that there could be a possible error in the sequence assembly. In a separate study Zhao et al. [25] used the SNP-based oligonucleotide array to map *dpy(s1272) *and found that the region corresponding to bhP18 is misassembled. Therefore, we have assigned bhP18, as well as *dpy(s1272) *and *unc(sa972)*, to the very end of the left arm of chromosome 3. Additional mapping using polymorphisms and visible markers will resolve the extent of misassembly.

In addition to the above BSA approach we also analyzed single F2 mutants segregating from a cross (termed single recombinant analysis or SRA) to determine recombination distances between polymorphisms and mutations. For this we used a multivulva mutation *lin(sy5353) *and an Unc mutation *unc(sy5506)*. The *lin(sy5353) *mutation is located on chromosome 1 since it is strongly linked to three small indels bhP1, bhP7 and bhP42 (B.P. Gupta, unpublished results). From a cross between *lin(sy5353) *and HK104 we picked 23 F2 mutant animals (46 chromosomes) and analyzed their DNA for the presence of bhP1. A single recombinant chromosome was recovered suggesting a recombination frequency of ~2% between *lin(sy5353) *and bhP1. In the case of *unc(sy5506) *mutation, located on chromosome X, we analyzed 20 F2 mutant animals for the presence of indel bhP26. A total of 4 recombinant chromosomes were recovered (see additional file 5) suggesting that the two loci are 10% apart. These results demonstrate that SRA mapping protocol can be used in *C. briggsae *to localize mutations to sub-chromosomal regions and narrow down their genetic interval.

## Discussion and Conclusions

We took a bioinformatics approach to identify polymorphisms in the *C. briggsae *genome and experimentally validated a small set of these to facilitate mapping of mutations. Comparison of AF16 (reference strain) to four other isolates (HK104, HK105, VT847 and PB800) revealed that HK104 is most polymorphic since it has the highest density of SNPs among all the strains. Altogether we identified ~31,300 polymorphisms (23,800 SNPs and 7,500 medium and small indels) between AF16 and HK104 that promise to be a valuable resource for mutation mapping and genome evolution studies. Roughly 20% of the SNPs are predicted to alter restriction enzyme sites (snip-SNPs) that could be detected by PCR followed by restriction digestion and agarose gel electrophoresis.

A total of 107 polymorphisms (20 snip-SNPs, 42 medium indels, and 45 small indels) that were experimentally tested, 66 (12 snip-SNPs, 22 medium indels, and 32 small indels) showed DNA fragments identical (or close) to *in silico *predictions (Table 5). Another 9 cases (2 snip-SNPs and 7 medium indels) were significantly different but nonetheless showed the presence of underlying variants. In 15 cases no polymorphism could be detected. Thus, excluding PCR failures (total 17), the success rate of correctly predicted polymorphisms was 73% (69-86% range) (Table 5). This suggests that both ssahaSNP and BreakPointRead algorithms work equally efficiently regardless of the type of polymorphism in question. A similar study in *C. elegans *[26] showed that greater than 95% of the polymorphisms predicted by the Polybayes program [27] are true. It remains to be seen whether the lower success rate in *C. briggsae *is due to intrinsic differences between the programs alone or if the quality of sequence data and assembly are additional contributing factors.

We used snip-SNPs and indels to map 12 mutations with visible phenotypes, and found that polymorphism-based mapping agreed with phenotypic marker-based results. Furthermore, it helped map two mutations, *lin(bh25) *and *unc(sy5415)*, for which no prior genetic linkage data was available. Five mutations were also localized to sub-chromosomal regions. Thus our mapping resource can be used to rapidly map new mutations in *C. briggsae*. It is also relatively easy to validate additional polymorphisms if one needs a greater resolution. It should be pointed out that Hillier et al. [6] have validated another set of 9 snip-SNPs by sequencing during the process of *C. briggsae *genome sequence assembly (see additional file 6). Given the high density of such markers (>2,000), it should be possible to map a mutation within a small genetic interval to facilitate molecular cloning (e.g., see [28]).

In addition to mapping mutations, SNPs and indels could also be used to improve the genetic linkage map of *C. briggsae*. The current *C. briggsae *sequence assembly, cb3, incorporates 90.2% (91.2 Mb) of the genome united into six chromosomes [6]. The remaining 9.8% of sequences are tentatively associated with chromosomes. These unmapped regions could be integrated into chromosomes by polymorphism-based recombination mapping. We have successfully used this approach to place the contig *fpc4184 *in the vicinity of *fpc3441 *(chromosome 1) based on the recombination distance of 5% between bhP42 (*fpc4184*) and bhP1 (*fpc3441*) (Figure 3) (A. Seetharaman, P. Cumbo, B. Nagagireesh and B. P. Gupta, manuscript submitted). In the other case, we have reassigned the bhP18 contig *fpc4010 *to the left arm of chromosome 3 based on its tight linkage to *dpy(s1272) *and *unc(sa972)*. Additional snip-SNPs and indels could further refine the locations of these contigs.

## Methods

### Strains and culture conditions

All strains were maintained at 22°C. The general methods of culturing nematodes are previously described [29]. AF16 is the wild type genetic background for all strains. The four other wild-type *C. briggsae *isolates that were used for polymorphism discovery are HK104, HK105, VT847, and PB800. The HK104 strain was used in all mapping experiments. Various mutations used in this study are: *csp-1(sa972), dpy(nm4), dpy(s1272), dpy(sy5001), dpy(sy5148), lev(sy5440), lin(bh20), lin(bh25), lin(sy5353), unc(sa997), unc(sy5415), unc(sy5422)*, and *unc(sy5506)*.

### In silico predictions of polymorphisms

#### SNPs

SNP discovery was performed on 13,632 shotgun sequence traces from strains HK104, VT847, HK105, and PB800. The ssahaSNP program (version SSAHA2) [24] was used to call SNPs due to its robust and efficient performance; only polymorphisms with quality scores above the minimum threshold were accepted. We also tested Polyphred (v5.04) [30] and PolyBayes (v3.0) [27] programs but found that only ssahaSNP could efficiently handle the entire read set and reference genome sequences as input. For a reference sequence the cb25 genome sequence assembly, which is based on strain AF16 and organized into ultra (fingerprint) contigs, was obtained from Wormbase. Flanking sequences for predicted SNPs were repeat-masked to lower case using the RepeatMasker program (v3.1.5) [31] with a customized *C. briggsae *repeat library.

The HK104 SNPs were positioned on the cb25 sequence assembly during SNP discovery. To position them on the newer cb3 sequence assembly, which is by chromosome, we obtained the assembly AGP files from Wormbase. SNP positions were inferred based on the coordinates and orientation of their cb25 ultracontig. SNPs on cb3-unmapped ultracontigs were mapped by WU-BLAST v2.0 (Gish, W., personal communication) alignment of their flanking sequences. Some 699 SNPs could not be positioned on the cb3 assembly by either method.

#### Indels

Candidate AF16-HK104 indels were extracted from HK104 sequence traces using the *parse_indel *utility of ssahaSNP. In the HK104 set, the largest indel event identified by ssahaSNP was 49 bp. To identify larger insertion/deletion variants we implemented BreakPointRead, a custom algorithm that detects structural variations (insertions, deletions, inversions, and copy number variants) spanned by individual sequence traces. Traces were aligned to the reference genome (cb25) using WU-BLAST v2.0 (Gish, W., personal communication), and screened for alignments with "gaps" of > = 10 bp. The alignment patterns of such "breakpoint reads" were used to infer the type and size of polymorphism. Predicted insertions and deletions were set aside for assay development.

#### bdP polymorphisms

The *bdP *polymorphisms described in this study (snip-SNP *bdP3 *and medium indels *bdP1 *and *bdP4*) were identified in the laboratory of SEB. The snip-SNP *bdP3 *was earlier used in a study involving ray pattern variation in *C. briggsae *[32].

### Development of RFLP and PCR Length Polymorphism (PLP) assays

SNPs were screened for substitutions that altered the recognition sequence of restriction enzymes using the *Bio::Restriction::Analysis *library of BioPerl [33]. The analysis was limited to 30 restriction enzymes from REBASE [23] known to be reliable and inexpensive. PCR assays were designed (amplicon sizes of 500 to 1000 bp, primer Tm's of 54-56 °C) using a local installation of the *primer3 *program [34]. *In silico *fragment analysis of the PCR products was performed to predict band sizes for AF16 and HK104; assays with more than 4 bands in either strain were removed.

In the case of small indels (7-49 bp), primers were selected to generate AF16 amplicon sizes within the range of 200 and 400 bp. For medium indels (50-2,000 bp), primers flanking each indel and specifying an AF16 amplicon size of 300-800 bp were selected.

### PCR

In all experiments the genomic DNA from F2 worms (derived from a cross between AF16 and HK104 animals) was used as a PCR template. In some control experiments genomic DNA from F1 heterozygous animals was also used. PCR results that gave rise to unexpected or no products were repeated at least twice. In some cases we also tested different annealing temperatures. Those that consistently failed were termed as "PCR failure".

For RFLP and medium indel assays, 10 ng dry PCR primers (IDT, Coralville, IA) were resuspended into 40 μM in a 96-well format. Our PCR mixture consisted of 300 pg genomic DNA, 0.1 μM PCR of each up and down primer, 0.02 U Platinum Taq (Invitrogen), 83.3 μM per base dNTPs, 2.92 μM MgCl_2_, 10× Buffer (16.7 mM Tris-HCl pH 8.4, 41.67 mM KCl - Invitrogen PCR Kit), and 4.2% DMSO. Amplification was carried out in a Perkin-Elmer 384 PCR plate containing 12 μl of 1× PCR mixture. The 384-well plate was sealed with Microseal A (MJ Research) before carrying out the PCR. After initial heat denaturation step (95°C - 2 min.) we used a fixed 35 cycles PCR (94°C - 10 sec., 58°C - 20 sec., 68°C - 30 sec.) and a final extension (68°C for 10 min.). PCR products were analyzed on 10% polyacrylamide gels that consisted of 33.3% of 29:1 acrylamide:bis (Biorad), 10% of 10×TBE, 15% of glycerol, 40.2% H_2_0, 3.4 × 10^-2^% TEMED (Int'l Biotech), and 1.42% of 10%APS. Gels were stained with SYBR green (Invitrogen) and inspected over a UV light box at 254 nm.

To detect small indels (<50 bp) we used a standard 35 cycles PCR (94°C - 10 sec., 48°C - 30 sec., 72°C - 60 sec.) for all amplifications. For BSA-based mapping the genomic DNA was prepared from 25 adults in a 10 μl lysis buffer (consisting of 50 mM KCl, 10 mM Tris pH 8.2, 2.5 mM MgCl, 0.45% Tween 20, 0.45% NP40, 0.001% Gelatin, and 30 μg Proteinase K). The mixture was frozen at -80°C (at least 30 min) and then placed in a thermal cycler for 1 hr incubation at 60°C followed by 15 min heat inactivation at 95°C. The resulting genomic DNA was diluted to 25 μl using sterile distilled water and stored at -20°C. The PCR mixture (25 μl) contained 1 μl genomic DNA, 1 μl each of up and down primers, 1 μl dNTPs, 2.5 μl NEB ThermoPol 10× PCR buffer, 0.2 μl NEB Taq Enzyme, and 18.3 μl sterile distilled PCR grade water. For SRA-based mapping, single worms were placed in 5 μl lysis buffer and processed as above. The lysed samples were used as DNA templates in PCR experiments. The amplified products were first analyzed on 1% agarose gel (Invitrogen UltraPure, Catalog #15510-027). Successful amplifications were subsequently examined on a 4% high-resolution agarose gel (Invitrogen UltaPure Agarose-1000, Catalog #10975-035) to determine the presence of indels.

### Mutation mapping

We picked 12 strains for linkage mapping studies (Table 6). The mutations were obtained from EMS (ethyl methane sulfonate) mutagenesis screens in an AF16 genetic background in various laboratories. The strains were outcrossed several times (3 or more). For mapping, mutant hermaphrodites were crossed with HK104 males and the genomic DNA from 20 F2 animals (wild type and mutant separately) was prepared as described in the previous section. The linkage was determined by PCR using protocols established for control experiments.

### Linkage and ULV analysis

To determine the linkage of a mutation to a chromosome, we initially relied on the visual inspection of DNA band intensities on Ethidium bromide-stained agarose gels. Subsequently, in indel-based mapping experiments, we calculated linkages as unitless values (ULVs) for an unbiased analysis. The mean intensities of DNA bands were measured by NIH ImageJ software (version 1.41o; [35]) using Measure tool under Analyze menu. For each mutation a ratio of band intensities in the "mutant" lane was calculated by dividing the mean intensities of the AF16 bands by the mean intensities of the HK104 bands. This ratio was termed as the ULV. As expected, ULVs were one for unlinked mutations and higher for linked mutations.

### Genetic positions of polymorphisms

The genetic positions of snip-SNPs and indels in this study correspond to nearest SNPs that were experimentally validated (D.C.K. and R.D.M., unpublished). These 'verified' SNPs (400 in total) were genotyped in RILs derived from two independent crosses between AF16, HK104 and VT847 (AF16 × HK104 and AF16 × VT847). The details are available on the *C. briggsae *SNP Research Facility website [36].

### Polymorphisms and sequence data availability

All identified snip-SNPs, indels and PCR primers, confirmed or otherwise, are accessible via the *C. briggsae *resource website (see "Polymorphism" link) [21]. The website also contains sequence reads of all four species (HK104, VT847, HK105, and PB800). The sequence directories are organized into Polyphred structure and contain additional files (such as read quality). Additional information on polymorphism discovery using sequence data can be obtained from the Washington University *C. briggsae *SNP Research Facility website [36]. The confirmed polymorphisms have also been submitted to Wormbase [37].

## List of abbreviations

BSA: Bulk segregant analysis; Indel: Insertion-deletion; PLP: PCR fragment length polymorphism; RIL: Recombinant inbred line; RFLP: Restriction fragment length polymorphism; SNP: Single nucleotide polymorphism; SRA: Single recombinant analysis; ULV: Unitless linkage value.

## Authors' contributions

The *C. briggsae *Advisory Group (BPG, ESH, HMC, RDM, and SEB) planned the project and collected the genetic and financial resources to perform the work. DCK and RDM carried out bioinformatics searches. DCK designed snip-SNP and PLP assays. JES, BT and KH performed PCR experiments to validate polymorphisms and map mutations. BT and BPG calculated ULVs for mutations linked to medium and small indels. bdP polymorphisms were identified and validated in the laboratory of SEB. BPG wrote the paper with input from ESH, HMC, SEB, DCK, and JES. All authors read and approved the final manuscript.

## Supplementary Material

Additional file 1 **List of SNPs in *C. briggsae***. An Excel file containing 23,829 computationally identified SNPs in AF16 and HK104. Click here for file

Additional file 2 **List of snip-SNPs and RFLP assays**. An Excel file containing 1988 SNPs that alter restriction enzyme site (snip-SNPs) along with PCR primers and predicted digestion patterns in AF16 and HK104. Click here for file

Additional file 3 **List of medium indels and PLP assays**. An Excel file containing 214 medium indels along with PCR primers and predicted DNA fragments in AF16 and HK104. Click here for file

Additional file 4 **List of small indels and PLP assays**. The Excel file containing 436 small indels along with PCR primers and predicted DNA fragments in AF16 and HK104. Click here for file

Additional file 5 **Mapping of *unc(sy5506) *mutation by single recombinant analysis (SRA)**. Twenty single F2 mutant animals were individually examined by PCR for the presence of indel bhP26. Four of these (#7, #10, #16, and #17) were found to be recombinants, as judged by the presence of two bands on the agarose gel (corresponding to AF16 and HK104 DNA). Click here for file

Additional file 6 ***C. briggsae *snip-SNPs identified prior to this work**. An Excel file containing nine *C. briggsae *snip-SNPs that were validated by Hillier et al. [6]. Click here for file
