# Supplementary figures and images for: A toolkit for rapid gene mapping in the nematode Caenorhabditis briggsae
Source: BMC Genomics. 2010 Apr 13;11:236. doi: 10.1186/1471-2164-11-236 (PMC2864247; doi:10.1186/1471-2164-11-236)

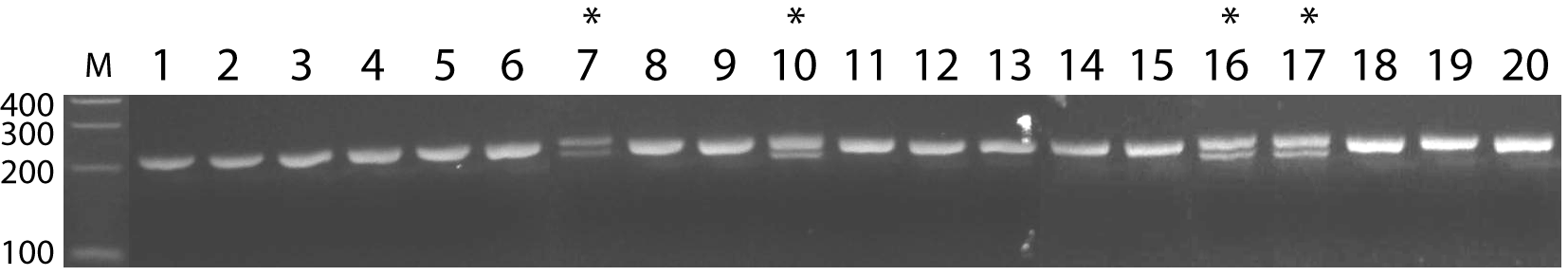

Supplement: Additional file 5 — Mapping of unc(sy5506) mutation by single recombinant analysis (SRA). Twenty single F2 mutant animals were individually examined by PCR for the presence of indel bhP26. Four of these (#7, #10, #16, and #17) were found to be recombinants, as judged by the presence of two bands on the agarose gel (corresponding to AF16 and HK104 DNA). [file 1471-2164-11-236-S5.PNG]
